# Intraspecific Variability in Leaf Functional Traits Reveals Divergent Resource-Use Strategies and Geographic Adaptation in Mediterranean Olive Cultivars from Worldwide Olive Germplasm Bank of Marrakech

**DOI:** 10.3390/plants15030471

**Published:** 2026-02-03

**Authors:** Jalal Kassout, Houda Souali, Asma Zahiri, Omar Abou-Saaid, Ater Mohammed, Sara Oulbi

**Affiliations:** 1 Regional Center of Agricultural Research of Marrakech, National Institute of Agricultural Research, Avenue Ennasr, BP 415, Rabat Principale, Rabat 10090, Morocco; houda.souali99@gmail.com (H.S.); asmazahirii03@gmail.com (A.Z.); omar.abousaaid@gmail.com (O.A.-S.); sara.oulbi@inra.ma (S.O.); 2 Centre d’Agrobiotechnologie et Bioingénierie, URL-CNRST 05, Université Cadi Ayyad, Marrakech 40010, Morocco; 3 Independent Researcher, Tétouan 93000, Morocco; mohammed.ater@gmail.com

**Keywords:** leaf traits, cultivated olive, intraspecific traits variability, functional responses, adaptation

## Abstract

Understanding intraspecific functional trait variability (ITV) is crucial for elucidating plant functional strategies under environmental change. This study investigates the functional responses of 129 Mediterranean olive (*Olea europaea* L.) cultivars conserved in the Worldwide Olive Germplasm Bank of Marrakech (WOGBM), focusing on three key leaf traits: specific leaf area (SLA), specific leaf water content (SLWC), and leaf area (LA). Substantial ITV was observed, with variability predominantly driven by cultivar differences and geographic origin. LA accounted for the highest within-cultivar variability (43.60%), followed by SLWC (31.67%) and SLA (17.92%). Geographic origin significantly influenced trait expression, with eastern Mediterranean cultivars exhibiting conservative resource-use strategies (high SLWC, low SLA, and LA), while western cultivars displayed acquisitive strategies (high SLA and LA, low SLWC). Principal component analysis further differentiated eastern and western cultivars, reflecting biogeographical and evolutionary influences. The relationship between LA and climatic variables suggests climate-driven selection, where cultivars from wetter regions develop larger leaves to optimize light capture and carbon assimilation, whereas those from drier environments exhibit smaller leaves to reduce water loss. These findings highlight a trade-off between resource acquisition and conservation, supporting the leaf economic spectrum at the intraspecific level. This study underscores the importance of ITV in olive diversification and adaptation, providing insights for breeding, conservation, and climate resilience. A trait-based approach proves valuable for exploring domestication processes and plant responses to environmental gradients.

## 1. Introduction

The olive tree (*Olea europaea* L.) is one of the most iconic and emblematic species of the Mediterranean region [1], holding exceptional cultural, economic, and ecological significance [2,3]. It belongs to the *Olea europaea* complex, which comprises six subspecies distributed across Asia, Africa, and Europe [1,4,5,6]. Among these, only *O. europaea* subsp. *europaea* has been domesticated [1], making it one of the first and oldest tree crops cultivated by humans in the Mediterranean basin, intricately linked to the ancient civilizations of the Near East [2,7]. The wild olive, or oleaster (*O. europaea* subsp. *europaea* var. *sylvestris*), is recognized as the ancestor of the cultivated olive (*O. europaea* subsp. *europaea* var. *europaea*), and both forms coexist in the Mediterranean region [1]. This coexistence, combined with regular gene flow between wild and cultivated forms, has resulted in complex and diverse genetic populations comprising domesticated, feral, and wild trees [8,9,10]. Genetic studies of oleaster populations using plastid DNA markers have identified three maternal lineages: E1, characteristic of the Eastern Mediterranean Basin, and E2 and E3, predominant in the Western Mediterranean [1,11]. Interestingly, most contemporary olive cultivars are affiliated with the E1 lineage from the Eastern Mediterranean region [1]. Paleobotanical, archeological, historical, and genetic studies [9,11,12,13] suggest multiple centers of olive domestication from autochthonous oleaster populations. These studies highlight the significant role of human activity and favorable climatic conditions in the expansion of olive cultivation across the Mediterranean [2,14,15]. Consequently, the diversity observed in olive germplasm reflects the empirical and localized selection of exceptional trees since the domestication of the olive in the Middle East approximately 6500 years ago [16]. Currently, more than 2000 olive cultivars exist, predominantly concentrated in Mediterranean countries such as Spain [17], Italy [18], France [19], and Portugal [20]. However, only a small subset of these cultivars (5–10%) is utilized in intensive farming systems, contributing to 99% of global olive production [21], while the remaining cultivars are closely associated with specific geographical regions [22,23,24].

Despite extensive research on the genetic diversity, biogeography, and domestication history of the olive tree, few studies have addressed its ecological and functional responses [12,22,24,25,26,27,28,29,30]. Trait-based approaches have proven effective in describing plant functional responses and identifying general adaptive strategies to environmental conditions [31,32,33,34]. Plant functional traits—physiological, morphological, or phenological characteristics—are directly linked to individual performance and serve as proxies for a plant’s capacity to acquire and utilize resources [35,36,37,38]. Studies of leaf trait variability across species have revealed a major axis of variability, known as the ’Leaf Economic Spectrum’ (LES), which describes a fundamental trade-off between rapid nutrient acquisition and resource conservation within well-protected tissues [31,32,39]. For example, traits such as specific leaf area (SLA) and leaf nitrogen content per area (N_area_) are associated with enhanced resource acquisition, while higher leaf dry matter content (LDMC) and specific leaf water content (SLWC) indicate resource allocation to denser tissues with greater water content [27,29,31,40,41]. Hence, the interrelationship between functional traits and environmental conditions has made it possible to understand plant species functional responses at multiple scales [27,41,42]. Although the LES has primarily been identified at the interspecific level [31], recent studies demonstrate that intraspecific trait variability (ITV) within single species is significant [27,29,43,44,45,46,47]. This variation has profound implications for climate vulnerability [28,48,49] and can differentiate co-occurring species [50]. Additionally, trait-based approaches are invaluable in evaluating the effects of crop selection and breeding on ecological performance under diverse conditions [24,51,52,53,54].

In this context, understanding functional differences within crop species like the cultivated olive is crucial for assessing domestication effects on crop diversity and adaptation. This knowledge is particularly relevant for selecting cultivars suited to current and future climatic conditions [55]. This study employs a trait-based approach to cultivated olives, integrating a comprehensive range of cultivars representative of the Mediterranean region and grown under similar conditions, to achieve two main objectives: (1) to explore the intraspecific variability in leaf functional traits and their dimensions in relation to cultivar origin and genetic characteristics, and (2) to evaluate how variations in these traits can illuminate the functional responses of cultivated olives depending on their biogeographical origin and, therefore, domestication history. Addressing these questions could provide a relevant basis for understanding the evolutionary and biogeographical history of cultivated Mediterranean olives.

## 2. Results

### 2.1. Overall Variation in Olive Leaf Traits Among Cultivars

**Figure 1:**
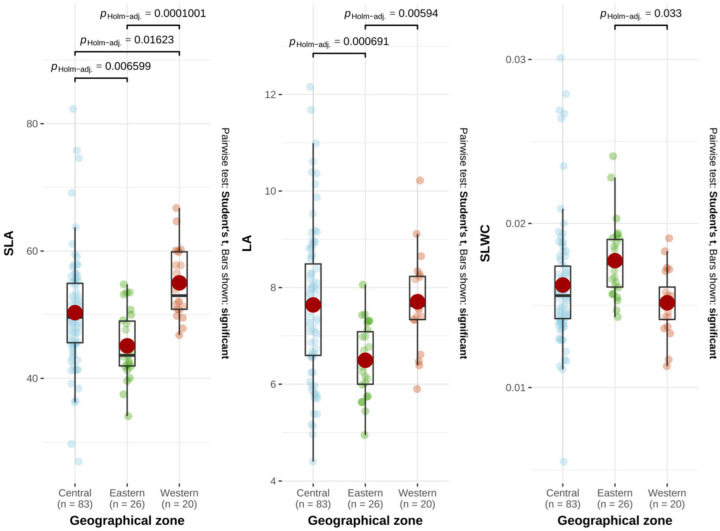
Variability in leaf traits according to geographic origin.

Significant differences in mean values were observed for all studied leaf traits across the olive cultivars (Table 1), with coefficients of variation (CV%) indicating substantial variability among traits (Supplementary Table S1). Leaf area (LA) exhibited the highest variance ratio (F = 7.28, *p* < 0.001), with mean values ranging from 4.40 cm^2^ in “Lastrino” to 12.16 cm^2^ in “Ogliarola del Bradano”. The CVs for LA ranged from 0.32% (“Morcone”) to 42.63% (“Minekiri”). Specific leaf water content (SLWC) ranged between 0.005 g H_2_O cm^−2^ (“Olivastra di Montalcino”) and 0.031 g H_2_O cm^−2^ (“Beldi”), with CVs spanning from 0.45% (“Lastrino”) to 48.06% (“Llorón de Ronda”). Specific leaf area (SLA) varied from 26.96 cm^2^ g^−1^ (“Beldi”) to 82.33 cm^2^ g^−1^ (“Fouji vert”), with CVs ranging from 0.24% (“Cerasuola”) to 52.95% (“Minekiri”) (Table 1, Supplementary Table S1). Pearson correlation analysis revealed significant relationships among traits (Supplementary Figure S1). SLWC correlated negatively with both SLA (−0.54) and LA (−0.20), while SLA showed a positive correlation with LA (0.34). One-way ANOVA by geographic zone showed significant differences in all three traits (Table 1). Tukey’s HSD test revealed significant differences in SLA between geographic zones, while LA showed differences between central and eastern zones and between eastern and western zones. For SLWC, significant differences were detected only between the western and eastern zones (Figure 1). SLA and LA exhibited significant differences when grouped by nuclear pool, while SLA was the only trait that differed significantly by maternal lineage (Table 1).

### 2.2. Intraspecific Variability in Olive Leaf Traits

The variance decomposition of functional leaf traits revealed notable contributions from different hierarchical levels, including cultivar, geographic zone, nuclear pool, and maternal lineage (Table 2). Cultivar differences accounted for 43.60% of LA variability, 31.67% of SLWC, and 17.92% of SLA. Geographic zone explained 10.77% of the total variance in LA, 9.30% in SLA, and 3.04% in SLWC, while maternal lineage accounted for minor proportions of the variance in LA (2.97%) and SLA (1.49%) (Table 2). Multivariate analysis of variance (MANOVA) indicated significant differences in leaf traits by geographic zone (Pillai trace = 0.201, F = 4.53, *p* < 0.001), but not by nuclear pool (Pillai trace = 0.056, F = 0.78, *p* = 0.631) or maternal lineage (Pillai trace = 0.040, F = 1.68, *p* = 0.173). Given geographic origin’s prominent contribution to trait variability, further multivariate analyses focused on this level.

### 2.3. Multivariate Relationships and Variability Across Cultivars by Geographic Origin

**Figure 2:**
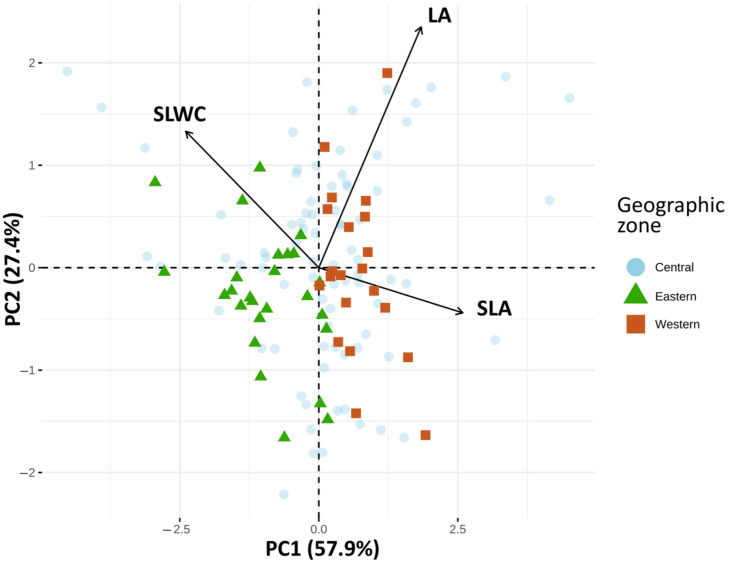
Principal component (PC) patterns of leaf functional traits of the studied olive cultivars.

Principal component analysis (PCA) of leaf traits showed that the first two axes explained 85.34% of the total variance (Figure 2, Table S2). The first axis (PC1), accounting for 57.93% of the variation, was positively correlated with SLA (0.858) and LA (0.610) and negatively correlated with SLWC (−0.793). The second axis (PC2), explaining 27.41%, was positively correlated with LA (0.779). Multivariate analysis identified distinct patterns among olive cultivars from the WOGBM based on geographic origin (Figure 2). PC1 clearly contributed in the discrimination between western Mediterranean cultivars (positive side of PC1) and eastern Mediterranean cultivars (negative side), with central Mediterranean cultivars distributed along both PC1 and PC2.

**Figure 3:**
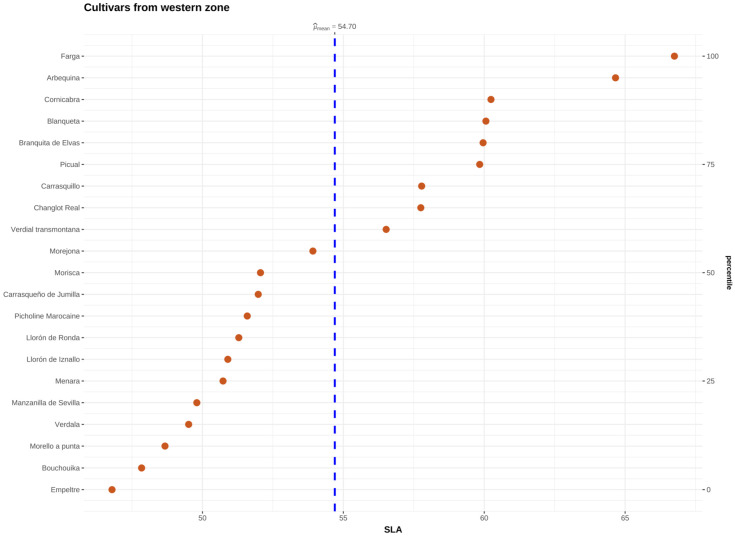
Distribution of olive cultivars depending on their SLA values.

Trait variability by geographic zone revealed the highest SLA for western cultivars in “Farga” (66.75 cm^2^ g^−1^) and the lowest in “Empeltre” (46.79 cm^2^ g^−1^), both from Spain (Figure 3). Among eastern cultivars, the highest SLWC was observed in “Abbadi Shalal” (0.024 g H_2_O cm^−2^), while “Djlot Tadmori” from Syria exhibited the lowest value (0.015 g H_2_O cm^−2^) (Supplementary Figure S2). For central Mediterranean cultivars, LA ranged from 4.40 cm^2^ in Lastrino to 12.16 cm^2^ in “Ogliarola del Bradano”, both from Italy. Among western cultivars, LA ranged from 5.9 cm^2^ in “Blanqueta” (Spain) to 10.22 cm^2^ in “Branquita de Elvas” (Portugal) (Supplementary Figure S3). Pairwise correlation analysis showed a negative relationship between SLA and SLWC in both eastern (−0.75) and central Mediterranean (−0.51) cultivars. SLA correlated positively with LA (0.36) only in central Mediterranean cultivars (Supplementary Figure S4).

Regarding the climatic conditions of the cultivars’ geographic origins, PCA using climatic variables revealed that the first principal component (PC1_Climat_) explained 69.2% of the total variation, representing a gradient of decreasing aridity (high MAP and AI) and increasing MAT and MAWM (Supplementary Figure S5). Accordingly, after performing the regression analysis (Supplementary Table S4), LA was negatively related with PC1_Climat_ (R^2^ = 0.216, *p* = 0.039) (Supplementary Table S4). Additionally, LA showed a negative relationship with MAWM (R^2^ = 0.309, *p* = 0.034).

### 2.4. Hierarchical Clustering Analysis of Olive Cultivars from the WOGBM

**Figure 4:**
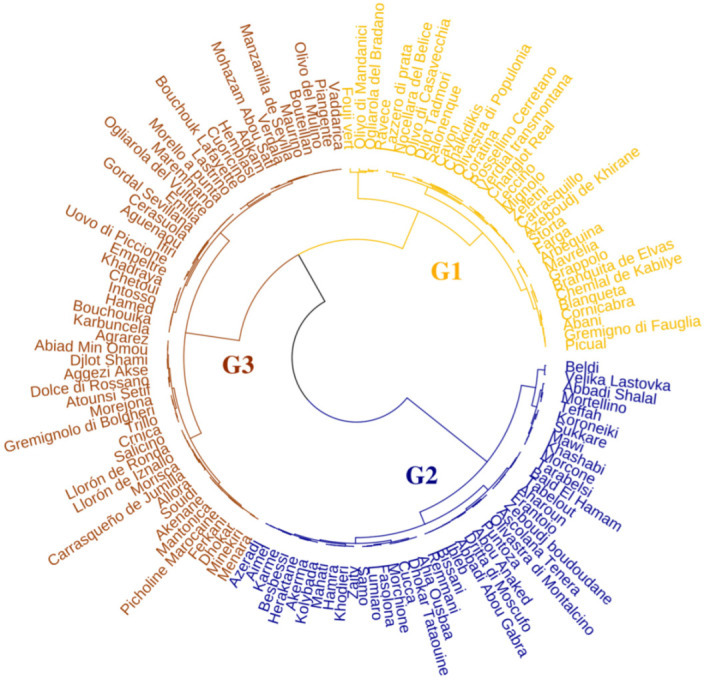
Hierarchical cluster analysis of the studied olive cultivars based on their functional traits (SLA, LA, and SLWC). The dendrogram illustrates the grouping of cultivars according to trait similarities, revealing distinct functional strategies.

Hierarchical clustering analysis identified three distinct groups of cultivars (Figure 4). Group 1 (G1) comprises 23 central Mediterranean cultivars, 9 western, and 1 eastern (“Djlot Tadmori” from Syria). This group comprises Italy (13 cultivars), Spain (7), Algeria (4), France (“Cayon”, “Salonenque”), Greece (“Chalkidikis”, “Mavrelia”), Portugal (“Branquita de Elvas”, “Verdial transmontane”), Slovenia (“Štorta”), Tunisia (“Fouji vert”), and Syria (“Djlot Tadmori”). Group 2 (G2) contains 26 central and 17 eastern Mediterranean cultivars, primarily from Syria (13), Italy (10), Algeria (8), Tunisia (3), Croatia (“Puntoza”, “Velika Lastovka”), Egypt (“Baid El Hamam”, “Hamra”), Greece (“Kolybada”, “Koroneiki”), Lebanon (“Bissani”, “Remmani”), and Slovenia (“Samo”). Group 3 (G3) encompasses 34 central, 11 western, and 8 eastern Mediterranean cultivars. Italy (20 cultivars) and Spain (8) dominate this group, along with contributions from Syria (6), Algeria (9), Morocco (“Picholine marocaine”, “Menara”, “Bouchouika”), Tunisia (“Chetoui”, “Dhokar”), Croatia (“Karbuncela”), Egypt (“Aggezi Akse”, “Hamed”), France (“Bouteillan”), and Slovenia (“Crnica”) (Supplementary Tables S1 and S3).

## 3. Discussion

Based on a comprehensive database of 129 olive cultivars from the Mediterranean region maintained at the WOGBM, this study aims to evaluate the utility of a trait-based approach to understanding the functional strategies of cultivated olive trees. The findings underscore the potential of this approach as a valuable tool for advancing research on olive breeding, adaptation, and genetic resource characterization. Examining functional trait variability within olive cultivars offers insights into the fundamental trade-offs shaping the functional responses of cultivated olives at the intraspecific level [29,34,47]. This approach also holds promise for elucidating the effects of selection and breeding on olive performance across diverse ecological conditions [24,51,52]. Using key functional traits, our results reveal substantial intraspecific variation among Mediterranean olive cultivars, reflecting distinct functional strategies. Leaf traits exhibited varying degrees of phenotypic variability depending on the cultivars’ geographic origins. Notably, despite being grown under similar conditions, the cultivars demonstrated distinct functional responses likely linked to their biogeographical and evolutionary history, besides the effect of local environmental conditions.

Analysis of olive leaf traits revealed significant differences among cultivars and high variation magnitudes (e.g., CV%), pointing to pronounced phenotypic variability and substantial cultivar differences [27,56]. This aligns with prior findings that highlight the considerable genetic and phenotypic variability among Mediterranean olive cultivars [57,58,59] and wild olives [27,28,29,60,61]. Significant differences in specific leaf area (SLA), specific leaf water content (SLWC), and leaf area (LA) among cultivars underscore divergent resource-use strategies [29,62]. SLA and LA, key traits within the leaf economic spectrum (LES), are strongly associated with resource-use efficiency [31,63], light interception, and thermal regulation during transpiration [64,65,66]. Conversely, SLWC enhances resource conservation, reducing vulnerability to stressful conditions such as drought [67,68,69]. Thus, previous studies have shown that leaf water content is crucial for thermal regulation and CO_2_ assimilation [40,70], which is vital under warmer and drier conditions. Moreover, SLWC was identified as a ‘mechanistic’ trait playing a key role in identifying mechanisms of climatic restriction in the olive tree [27]. Meanwhile, it is important to note that SLA and LA are highly plastic traits influenced by environmental factors such as temperature and water availability [71,72]. This plasticity has been previously documented for wild olive populations along aridity gradients in Morocco [29]. While the common garden experiment controls for environmental variability and allows for the detection of genetic differences among cultivars, it does not fully reflect SLA and LA expression in the cultivars’ native environments. As a result, olive cultivars may exhibit different trait values and varying degrees of plasticity, enabling them to adjust their functional responses to local environmental conditions.

The negative correlation observed between SLWC and both SLA and LA indicates a trade-off between resource acquisition (represented by SLA and LA) and resource conservation (assessed by SLWC) [27,31,70]. Furthermore, significant differences in these traits were detected based on the geographic origin of the cultivars (Figure 1). In addition, LA and SLA showed significant variability depending on the nuclear pool of the cultivars, while SLA was the only trait significantly different among cultivars with distinct maternal lineages (Table 1). Given that all cultivars were grown under the homogeneous environmental conditions of the WOGBM, the observed variation likely reflects the biogeographical and evolutionary history of the cultivars, as inferred from plastid haplotypes (maternal lineage) and nuclear microsatellite markers, shaped by adaptive processes [26,29]. The substantial variability observed in the studied leaf traits highlights the capacity of olive cultivars to respond to stressful environmental conditions [29]. This variability could also provide insights into the long-term adaptation of cultivars to the diverse environments of their geographic origins [26] (Supplementary Figure S5). In addition, the relationship found between LA and climatic variables further supports the role of climate-driven selection in shaping leaf trait variability among olive cultivars (Supplementary Table S4). The positive relationship observed between leaf area and decreasing aridity suggests that cultivars from wet regions tend to develop larger leaves, likely to optimize light capture and carbon assimilation under favorable growing conditions [73]. This pattern aligns with the well-documented influence of wet conditions on leaf size development [73]. Meanwhile, in drier conditions, trees may adopt alternative strategies and tend to develop small leaves with increased leaf thickness or reduced transpiration rates, to mitigate water loss [29,73]. These findings highlight the role of climatic selective pressures in shaping leaf functional traits and suggest that temperature-driven variation in LA could be an adaptive response to local environmental conditions.

Trait variance decomposition revealed significant trait variability, primarily driven by differences between cultivars. LA exhibited the highest variability (43.60%), followed by SLWC (31.67%) and SLA (17.92%). Geographic origin contributed to variability in SLWC (3.04%), SLA (9.30%), and LA (10.77%). However, contributions from nuclear pools and maternal lineage were minimal, accounting for only 1.49% and 2.97% of SLA and LA variability, respectively, or being negligible for other traits (Table S2). These findings are consistent with prior studies highlighting the contribution of ITV to total trait variability within species [29,44,45,47]. The significant proportion of variability at the cultivar level underscores the importance of within-individual variability, as previously documented [29,43]. Moreover, the variability in these traits along environmental gradients [27,31] likely explains the prominent role of geographic origin in total trait variability [47,74]. ITV plays a critical role in ecological and evolutionary processes, including species resilience and adaptation to stressors like aridity [48,75,76]. Exploring ITV in olive cultivars can deepen our understanding of mechanisms underlying olive diversification and functional responses to stress [24,26]. This is particularly relevant in the context of climate change, where such insights are crucial for improving cultivar resilience and adaptability [55].

The variance decomposition results indicate that the geographic origin of the studied olive cultivars is the primary driver of trait variability, and the findings of the principal component analysis (PCA) further corroborate this. The first PCA axis, which explains 57.93% of the variability, is positively associated with SLA and LA and negatively with SLWC, which highlights a clear distinction between cultivars from the western and eastern Mediterranean regions (Figure 2). The negative correlation between SLWC and both SLA and LA reflect a trade-off between acquisition and resource conservation at the intraspecific level in Mediterranean cultivars, consistent with previous findings for wild olive populations [27,29]. This relationship suggests a divergence in resource-use strategies. For instance, western cultivars, such as “Farga” and “Arbequina”, exhibited high SLA (Figure 3), indicative of rapid resource acquisition and vegetative growth under favorable conditions. Conversely, eastern cultivars, such as “Abbadi Shalal” and “Sukkare”, displayed low SLA and high SLWC (Figure S2), which are traits associated with enhanced water retention and resource conservation, allowing them to better withstand drier or stressful conditions [38,67,68].

This distinction in functional traits likely reflects the biogeographic history of olive domestication, which began in the Levant (eastern Mediterranean) around 6500 years ago [16]. Selected forms were progressively disseminated to central and western Mediterranean regions [1,77]. Human selection for desirable agronomic traits likely drove the development of functional strategies tailored to local environmental conditions and agricultural practices [78]. The introduction of eastern cultivars to western regions, where they likely hybridized with local oleaster populations [1], further contributed to phenotypic diversification and functional trait variation [24]. Genetic studies support this geographic differentiation, revealing significant contributions of western oleasters to cultivated olives in central and western Mediterranean regions [9,11]. The relatively recent origin of western cultivars [11] could explain their distinct functional profiles compared to their eastern counterparts (Figure 2). The western cultivars’ conservative strategies may reflect selection for traits that promote nutrient conservation in resource-poor habitats, shaped by less developed agricultural practices. In contrast, eastern cultivars’ more acquisitive strategies may be tied to their origins in regions with advanced cultural practices, such as irrigation, and nutrient-rich environments [25,38]. These findings underscore the adaptation signature associated with geographic zones of origin in cultivated olives [1], aligning with the concept of local adaptation. Meanwhile, integration of additional traits could further refine our understanding of olive cultivar differentiation and functional responses. The grouping of cultivars based on geographic origin and genetic background provides a broad perspective on trait variation, but it may overlook finer-scale differences within groups. Despite these limitations, the selected traits effectively capture major functional trade-offs and adaptive strategies among cultivars. Future research could expand trait selection to include additional functional traits, providing deeper insights into cultivar adaptation to diverse environmental conditions. Finally, the obtained results demonstrate the potential of trait-based approaches to elucidate olive domestication and diversification patterns in the Mediterranean region. Moreover, it contributes to ongoing research on plant functional responses to climate change, highlighting how trait variability can inform the development of resilient cultivars in a changing climate.

## 4. Materials and Methods

### 4.1. Plant Material

This study was conducted at the World Olive Gene Bank of Marrakech (WOGBM), managed by the National Institute of Agricultural Research (INRA), located at the Tessaout research station (31.819° N, −7.433° W, 618 m a.s.l.), approximately 60 km northeast of Marrakech, Morocco. The site is characterized by a semi-arid climate, with a mean annual temperature of 18.84 °C and average annual rainfall of 257 mm (1972–2019, [79]). A total of 129 cultivated *Olea europaea* L. cultivars from 13 Mediterranean countries (Supplementary Table S1) were evaluated. The cultivars were planted in clay loam soil at a 7 × 4 m spacing and managed under uniform agronomic practices, including drip irrigation, pruning, and fertilization. The WOGBM collection was established through gradual introductions between 2003 and 2012. The sample trees were in good health and represent the typical growth forms of each cultivar. All trees were carefully chosen to reflect the natural growth status of their respective cultivars, ensuring that they were not affected by external stress factors (e.g., disease, poor soil conditions, or mechanical damage). Based on nuclear genetic markers [11,57,80] and cytoplasmic markers [1,9], the cultivars were classified into Eastern, Western, and Central nuclear genepools, in addition to an Admixed gene pool (e.g., without clear affiliation to a distinct geographical gene pool), and into Eastern or Western maternal lineages (Supplementary Table S1). Additionally, the cultivars were grouped according to their geographic zone of origin: Western, Central, and Eastern Mediterranean [81]. Variability in functional traits was analyzed at four hierarchical levels: (1) cultivars—variation among different cultivars; (2) nuclear gene pool—variation among genetic pools; (3) maternal lineage—variation by cytoplasmic genetic markers, and (4) geographic zone—variation among the geographic zones of origin. Additionally, to relate the climatic conditions of the origin region of each cultivar, we used five climatic variables (Supplementary Table S1): mean annual temperature (MAT, °C), minimum temperature of the coldest month (MACM, °C), maximum temperature of the warmest month (MAWM, °C), and mean annual precipitation (MAP, mm) (Supplementary Table S1). The climatic data were extracted from the CHELSA database, at a resolution of approximately 1 km^2^ [82], to which the global aridity index (AI, unitless) was added from the CGIAR (Consortium of International Agricultural Research Centers) Global Aridity database [83]. These variables were chosen because they reflect long-term environmental conditions that shape plant adaptation and trait expression in a long-lived species such as the olive tree. Previous studies have identified these parameters as key drivers of variability in olive leaf traits, hydraulic strategies, and potential distribution patterns across Mediterranean landscapes [26,27,30]. While climate variability metrics (e.g., seasonality of temperature and precipitation) could provide additional insights, our study prioritizes long-term climatic averages, as they better capture the persistent resource availability that influences functional trait adaptation in olive cultivars over time.

### 4.2. Leaf Functional Traits

Leaf sampling and trait measurements followed standardized protocols [62]. Twigs with fully expanded, mature, and healthy leaves were collected from the sunlit upper canopy of each tree. Samples were wrapped in moist paper towels, sealed in plastic bags, and stored in a cooled isothermal box [84]. Forty-five healthy leaves were randomly selected from each tree for trait assessments. Two trees per cultivar were evaluated, making a total of 258 trees and 90 leaves sampled, representing each studied cultivar and resulting in a total of 11,610 replicate leaves. Following Kassout et al. [27,29], leaf fresh mass (LFM, g) was measured using an electronic balance. Leaf area (LA, cm^2^) was determined by scanning the leaves and analyzing the images in ImageJ software v 1.53e [85]. Leaves were then oven-dried at 70 °C for 72 h to measure leaf dry mass (LDM, g). Specific leaf area (SLA, cm^2^ g^−1^) was calculated as the ratio of total leaf area to total dry mass, while specific leaf water content (SLWC, g H_2_O cm^−2^) was calculated as the ratio of total leaf water content to total area [27]. The selection of three functional traits (SLA, LA, and SLWC) in our study was based on their widespread use in trait-based research aimed at understanding plant functional strategies [64,70]. These traits have been recognized as key indicators of resource-use strategies, particularly in relation to the leaf economics spectrum and plant responses to environmental constraints. For instance, Pierce et al. [86] demonstrated that using just three traits—SLA, LA, and lead dry matter content (LDMC)—effectively captured major axes of functional variation across diverse plant species and biomes at a global scale. This highlights the feasibility of using a minimal yet ecologically meaningful set of traits to characterize plant functional responses comprehensively. Moreover, the selected traits are easily measurable and highly informative, allowing us to maximize the scope of our study by analyzing a large number of olive trees (258 trees). Given the logistical constraints and the need to maintain methodological consistency across a large-scale sampling effort, integrating additional traits was beyond the scope of this study. However, we acknowledge that expanding trait coverage could provide further insights and should be considered in future research.

### 4.3. Statistical Analysis

All statistical analyses were conducted using R software v 4.3.1 [87] (R Development Core Team, 2017). To summarize leaf trait datasets, means and standard deviations were calculated at the cultivar, geographic zone, nuclear gene pool, and maternal lineage levels. Data obtained from two tree replicates of each cultivar were pooled to obtain a representative mean value per cultivar. The coefficient of variation (CV) was calculated as CV(%) = (standard deviation_trait_/mean_trait_) × 100 to quantify trait variability. Trait differences among cultivars were assessed using one-way ANOVA. Separate ANOVAs were conducted to test for differences by geographic zone and nuclear gene pool, with Tukey’s HSD post hoc test (*p* = 0.05) performed using the *agricolae* package [88]. Normality and homoscedasticity of the data were verified using Shapiro–Wilk and Levene tests, respectively. Differences between maternal lineages (East vs. West) were analyzed using unpaired *t*-tests. Multivariate analysis of variance (MANOVA) was used to evaluate simultaneous trait responses across multiple hierarchical levels, employing the *stats* package in R. Pearson correlations were computed using the *cor* function in R to investigate relationships between traits. To quantify the extent and relative contribution of variability at each hierarchical level [29], nested ANOVA with variance partitioning was performed using a general mixed model fitted with the restricted maximum likelihood (REML) method in the *nmle* package [89]. Variance components were extracted with the *varcomp* function in the *ape* package [90]. Principal component analysis (PCA) was conducted using the *FactoMineR* package [91] to explore differences among cultivars and trait syndromes and to assess the possible effect of the climate of the geographic origin of each cultivar on the studied functional traits. Hierarchical cluster analysis (HCA) was performed using Ward linkage and Euclidean distances, implemented in the *dendextend* [92] and *vegan* [93] packages. Robust linear regression was used to describe the relationship between olive functional traits and climatic variables, using the *robustbase* package [94].

## 5. Conclusions

This study highlights the substantial intraspecific variability in leaf functional traits among Mediterranean olive cultivars, reflecting divergent resource-use strategies and adaptive responses to geographic origins. The significant differences in SLA, SLWC, and LA between eastern and western cultivars, coupled with the negative correlations among these traits, underscore a trade-off between resource acquisition and conservation strategies. These findings align with the biogeographical and evolutionary history of olive domestication and diversification, suggesting that human selection and local environmental conditions have shaped the functional strategies of cultivated olive trees. Moreover, the demonstrated utility of a trait-based approach offers valuable insights into the ecological and adaptive mechanisms underlying olive diversification, contributing to the understanding of cultivar resilience and functional responses to climate change. These results emphasize the relevance of functional trait variability for breeding programs and the conservation of olive genetic resources in a changing environment.

## Figures and Tables

**Table 1:** Summary statistics of the studied leaf traits according to the studied hierarchical levels.

	SLWC (g H_2_O cm^−2^)	LA (cm^2^)	SLA (cm^2^ g^−1^)
All cultivars, *n* = 129 ^1^	0.016 ± 0.004 ^2^	7.42 ± 1.74	49.84 ± 12.63
*ANOVA F*	3.611 ***	7.284 ***	2.636 ***
**Geographical zone**			
Central, *n* = 83	0.016 ± 0.004 ^a,b^	7.640 ± 1.544 ^a^	50.30 ± 8.70 ^b^
Eastern, *n* = 26	0.018 ± 0.002 ^a^	6.493 ± 0.750 ^b^	45.10 ± 5.28 ^c^
Western, *n* = 20	0.015 ± 0.002 ^b^	7.706 ± 0.990 ^a^	55.00 ± 5.64 ^a^
*ANOVA F*	3.497 *	7.729 ***	9.463 ***
**Nuclear pool**			
Admixed, *n* = 50	0.016 ± 0.004 ^a^	7.371 ± 1.431 ^a,b^	49.40 ± 7.99 ^a,b^
Central, *n* = 34	0.016 ± 0.004 ^a^	7.853 ± 1.539 ^a^	52.09 ± 8.65 ^a^
Eastern, *n* = 31	0.017 ± 0.003 ^a^	6.804 ± 1.217 ^b^	46.92 ± 8.48 ^b^
Western, *n* = 14	0.016 ± 0.002 ^a^	7.901 ± 0.941 ^a,b^	53.79 ± 4.25 ^a^
*ANOVA F*	1.093 n.s.	3.818 *	3.459 *
**maternal lineage**			
East, *n* = 116	0.017 ± 0.004 ^a^	7.414 ± 1.453 ^a^	49.51 ± 8.23 ^a^
West, *n* = 13	0.015 ± 0.002 ^a^	7.466 ± 1.050 ^a^	54.21 ± 7.03 ^b^
*Student t-test*	−3.266 n.s.	0.162 n.s.	2.245 *

^1^ Number of cultivars. ^2^ Mean ± SD values. * *p* < 0.5. *** *p* < 0.001. n.s. non-significant. Values with the same suffix are not statistically significantly different at *p* < 0.05 in Tukey HSD post hoc tests.

**Table 2:** The variance decomposition (%) of the studied traits according to considered hierarchical level.

Traits	SLA	LA	SLWC
Geographical zone	9.30	10.77	3.04
Nuclear pool	0.00	0.00	0.00
Maternal lineage	1.49	2.97	0.00
Cultivars	17.92	43.60	31.67
Residual	71.29	42.66	65.29

## Data Availability

Data are contained within the article and Supplementary Materials.

## Supplementary Materials

The following supporting information can be downloaded at https://www.mdpi.com/article/10.3390/plants15030471/s1, Figure S1: Pearson correlation analysis between the studied functional traits; Figure S2: Distribution of olive cultivars depending on their SLWC values; Figure S3: Distribution of olive cultivars depending on their LA values; Figure S4: Pearson correlation analysis between the studied functional traits according to their geographic origin; Figure S5: Biplot of the principal component analysis (PCA) of climatic variables of the studied cultivars according to their main cultivation area and grouped by geographic origin; Table S1: List of 129 cultivars used in the present study classified according to their geographic origin, maternal lineage, and nuclear pool. Mean values of studied traits are provided (Mean ± SD), as well as the climatic variables of the geographic origin of each cultivar. A cluster group from hierarchical clustering analysis is provided; Table S2: Trait loading, eigenvalues, and percentage of trait variation explained by first three principal components (PCs); Table S3: The distribution of the studied cultivars after the hierarchical clustering analysis according to the main cultivation area, geographical zone, maternal lineage, and nuclear pool; Table S4: A summary of the robust linear regression analyses between leaf functional traits and climatic variables.
