# Supplementary material for: Intraspecific Variability in Leaf Functional Traits Reveals Divergent Resource-Use Strategies and Geographic Adaptation in Mediterranean Olive Cultivars from Worldwide Olive Germplasm Bank of Marrakech
Source: Plants (Basel). 2026 Feb 3;15(3):471. doi: 10.3390/plants15030471 (PMC12899711; doi:10.3390/plants15030471)
Supplement: Supplementary file 1 [file plants-15-00471-s001.zip › Table S2.docx]

Table S2. ﻿Trait loading, eigenvalues, and percentage of trait variation explained by the first three principal components (PCs).

| Traits | PC1 | PC2 |
| --- | --- | --- |
| SLA | **0.858** | -0.146 |
| LA | **0.610** | **0.778** |
| SLWC | **-0.793** | 0.441 |
| Eigenvalues | 1.738 | 0.822 |
| % of variance | 57.93 | 27.41 |
| Cumulative variance | 57.93 | 85.34 |

﻿values greater than 0.5 are in bold
