# Supplementary material for: Intraspecific Variability in Leaf Functional Traits Reveals Divergent Resource-Use Strategies and Geographic Adaptation in Mediterranean Olive Cultivars from Worldwide Olive Germplasm Bank of Marrakech
Source: Plants (Basel). 2026 Feb 3;15(3):471. doi: 10.3390/plants15030471 (PMC12899711; doi:10.3390/plants15030471)
Supplement: Supplementary file 1 [file plants-15-00471-s001.zip › Table S3.docx]

Table S3. Distribution of the studied cultivars after the hierarchical clustering analysis according to the main cultivation area, geographical zone, maternal lineage and nuclear pool.

|  | Group 1 (N = 33^a^) | Group 2 (N = 43) | Group 3 (N = 53) |
| --- | --- | --- | --- |
| Main Cultivation area | | | |
| Algeria ^C^ | 4 (12%)^b^ | 8 (19%) | 9 (17%) |
| Croatia ^C^ | 0 (0%) | 2 (4.7%) | 1 (1.9%) |
| Egypt ^E^ | 0 (0%) | 2 (4.7%) | 2 (3.8%) |
| France ^C^ | 2 (6.1%) | 0 (0%) | 1 (1.9%) |
| Greece ^C^ | 2 (6.1%) | 2 (4.7%) | 0 (0%) |
| Italy ^C^ | 13 (39%) | 10 (23%) | 20 (38%) |
| Lebanon ^E^ | 0 (0%) | 2 (4.7%) | 0 (0%) |
| Morocco ^W^ | 0 (0%) | 0 (0%) | 3 (5.7%) |
| Portugal ^W^ | 2 (6.1%) | 0 (0%) | 0 (0%) |
| Slovenia ^C^ | 1 (3.0%) | 1 (2.3%) | 1 (1.9%) |
| Spain ^W^ | 7 (21%) | 0 (0%) | 8 (15%) |
| Syria ^E^ | 1 (3.0%)^c^ | 13 (30%) | 6 (11%) |
| Tunisia ^C^ | 1 (3.0%)^d^ | 3 (7.0%) | 2 (3.8%) |
| Geographical zone | | | |
| Central | 23 (70%) | 26 (60%) | 34 (64%) |
| Eastern | 1 (3.0%)^c^ | 17 (40%) | 8 (15%) |
| Western | 9 (27%) | 0 (0%) | 11 (21%) |
| Maternal lineage | | | |
| East | 26 (79%) | 41 (95%) | 49 (92%) |
| West | 7 (21%) | 2 (4.7%) | 4 (7.5%) |
| Nuclear pool | | | |
| Admixed | 11 (33%) | 14 (33%) | 25 (47%) |
| Central | 13 (39%) | 11 (26%) | 10 (19%) |
| Eastern | 4 (12%) | 18 (42%) | 9 (17%) |
| Western | 5 (15%) | 0 (0%) | 9 (17%) |

^a^ Total number of cultivars per group from the hierarchical cluster analysis, ^b^ number and percentage of cultivars presented by each cluster and depending on the studied level. ^c^ Djlot Tadmori *cv.*, ^d^ Fouji vert *cv*.

^C^ Central, ^E^ Eastern, ^W^ Western.
