# Supplementary material for: Intraspecific Variability in Leaf Functional Traits Reveals Divergent Resource-Use Strategies and Geographic Adaptation in Mediterranean Olive Cultivars from Worldwide Olive Germplasm Bank of Marrakech
Source: Plants (Basel). 2026 Feb 3;15(3):471. doi: 10.3390/plants15030471 (PMC12899711; doi:10.3390/plants15030471)
Supplement: Supplementary file 1 [file plants-15-00471-s001.zip › Table S4.docx]

Table S4. Summary of the robust linear regression analyses between leaf functional traits and climatic variables.

|  |  | PC1_climat_^a^ | PC2_climat_^a^ | AI | MAP | MAT | MAWM | MACM |
| --- | --- | --- | --- | --- | --- | --- | --- | --- |
| SLA | R² | 0.132 | 0.006 | 0.091 | 0.142 | 0.041 | 0.077 | 0.016 |
|  | *p*-value | 0.153 | 0.815 | 0.265 | 0.157 | 0.492 | 0.343 | 0.684 |
|  | Relationship | n.s | n.s | n.s | n.s | n.s | n.s | n.s |
| LA | R² | 0.216 | 0.278 | 0.144 | 0.137 | 0.096 | 0.309 | 0.005 |
|  | *p*-value | **0.039** | 0.106 | 0.159 | 0.183 | 0.319 | **0.034** | 0.850 |
|  | Relationship | **-** | n.s | n.s | n.s | n.s | **-** | n.s |
| SLWC | R² | 0.024 | 0.014 | 0.012 | 0.040 | 0.022 | 0.040 | 0.003 |
|  | *p*-value | 0.484 | 0.596 | 0.621 | 0.355 | 0.497 | 0.361 | 0.821 |
|  | Relationship | n.s | n.s | n.s | n.s | n.s | n.s | n.s |

^a^ retrieved from the principal component analysis of climate variables. Traits and climate variable abbreviations are given in Material and Methods section. n.s. non-significant relationship. Bold values represent significant relationship.
