# Supplementary figures and images for: Intraspecific Variability in Leaf Functional Traits Reveals Divergent Resource-Use Strategies and Geographic Adaptation in Mediterranean Olive Cultivars from Worldwide Olive Germplasm Bank of Marrakech
Source: Plants (Basel). 2026 Feb 3;15(3):471. doi: 10.3390/plants15030471 (PMC12899711; doi:10.3390/plants15030471)

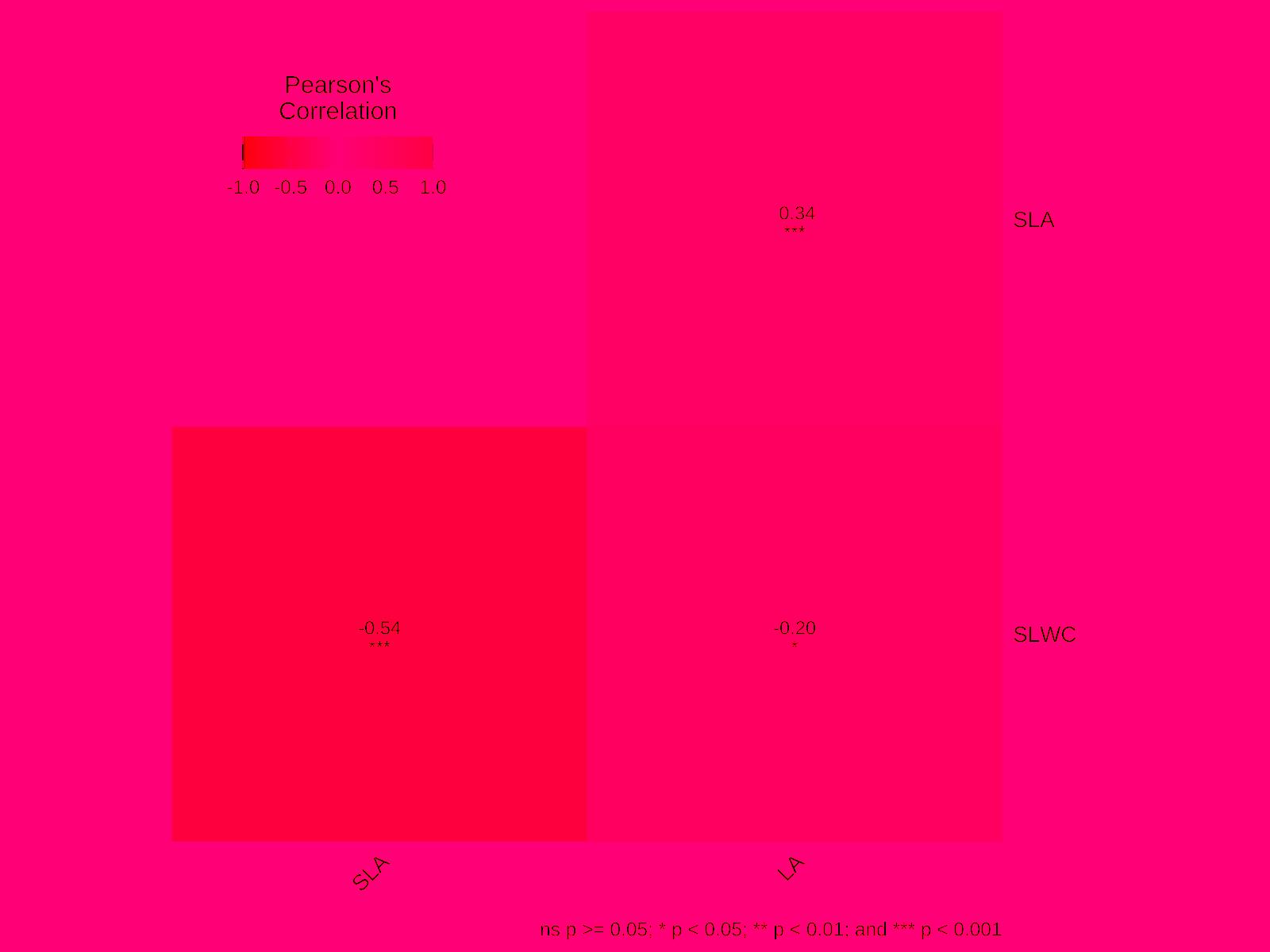

Supplement: Supplementary file 1 [file plants-15-00471-s001.zip › Figure S1.jpg]

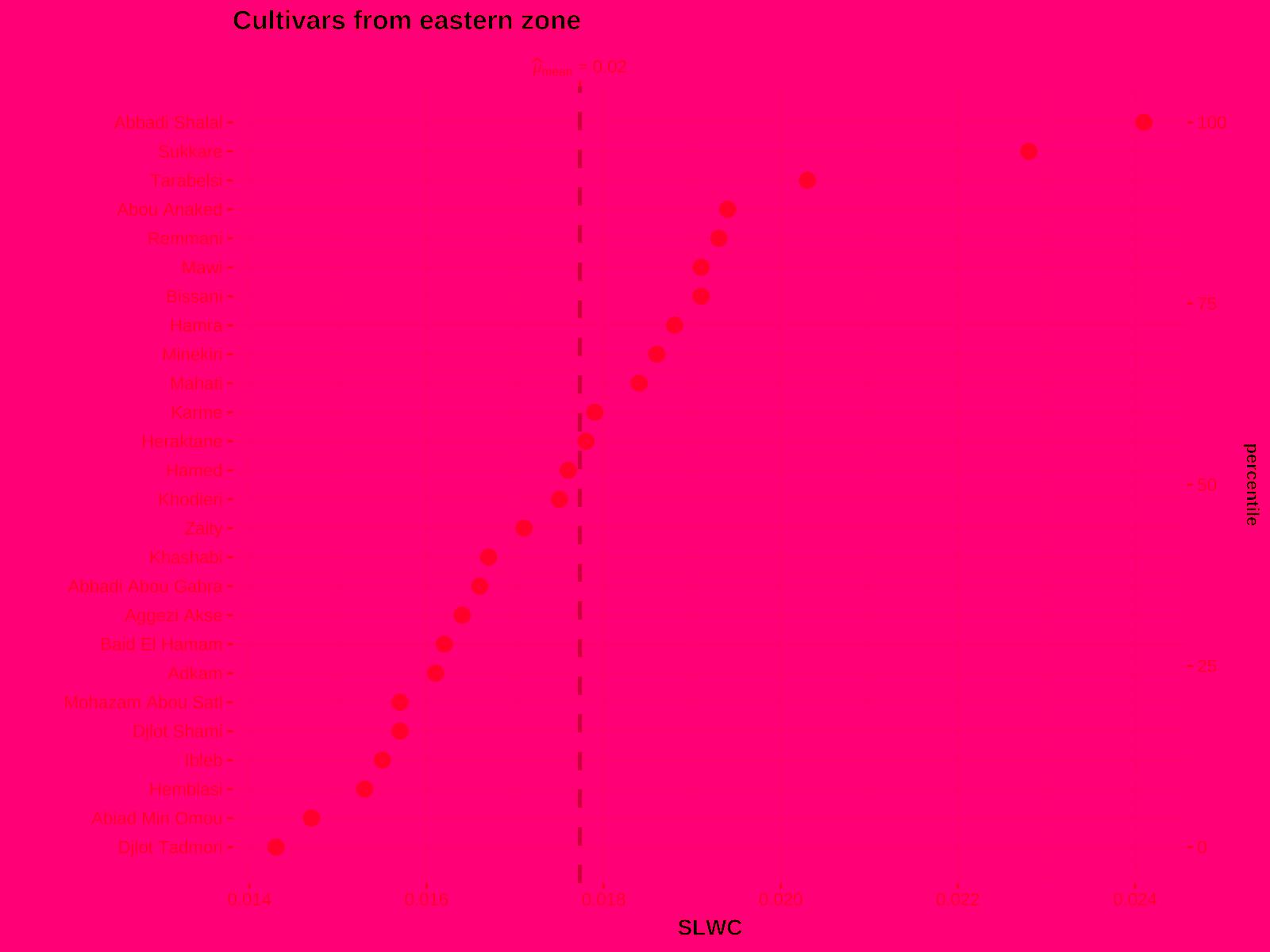

Supplement: Supplementary file 1 [file plants-15-00471-s001.zip › Figure S2.jpg]

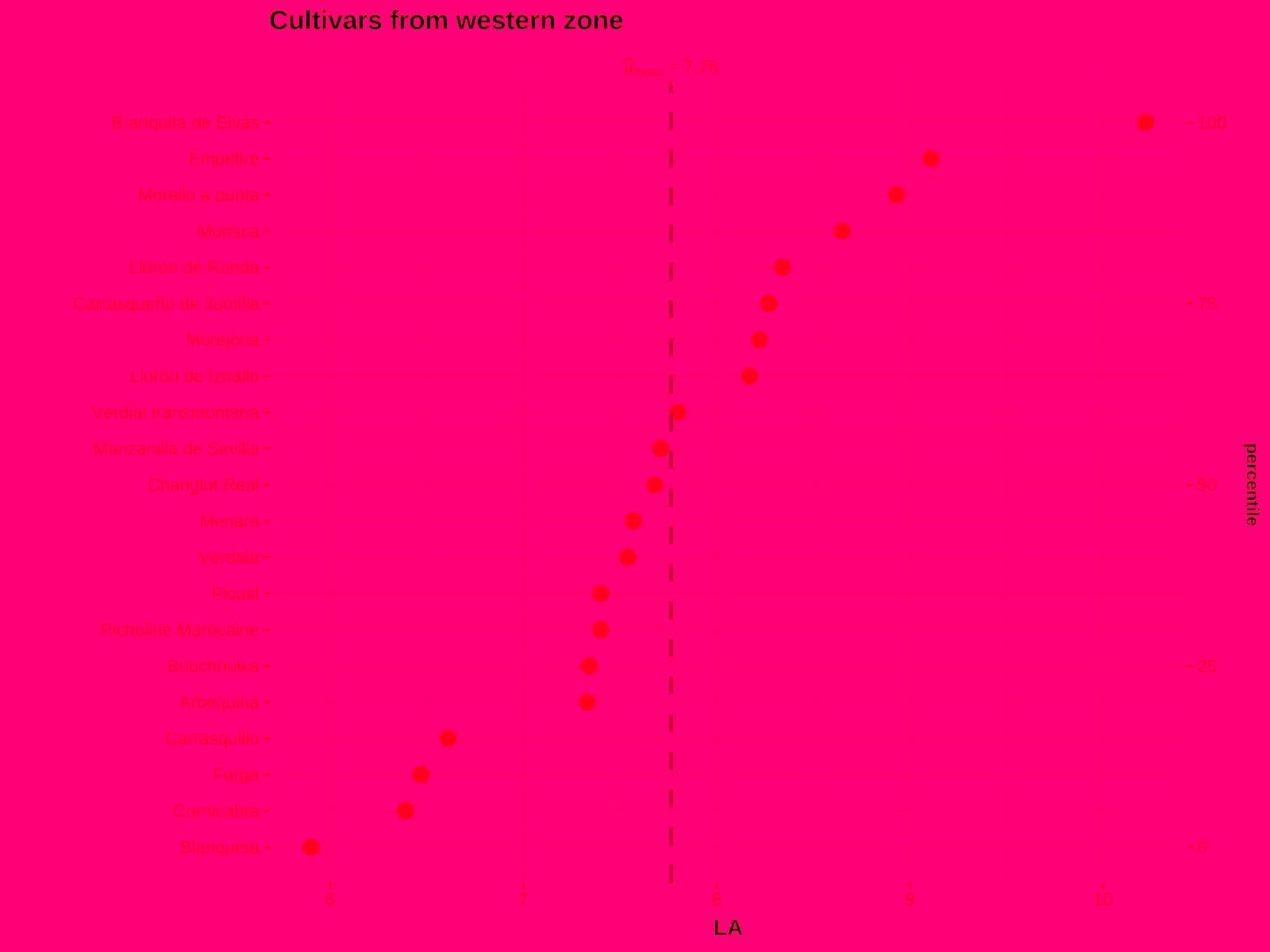

Supplement: Supplementary file 1 [file plants-15-00471-s001.zip › Figure S3.jpg]

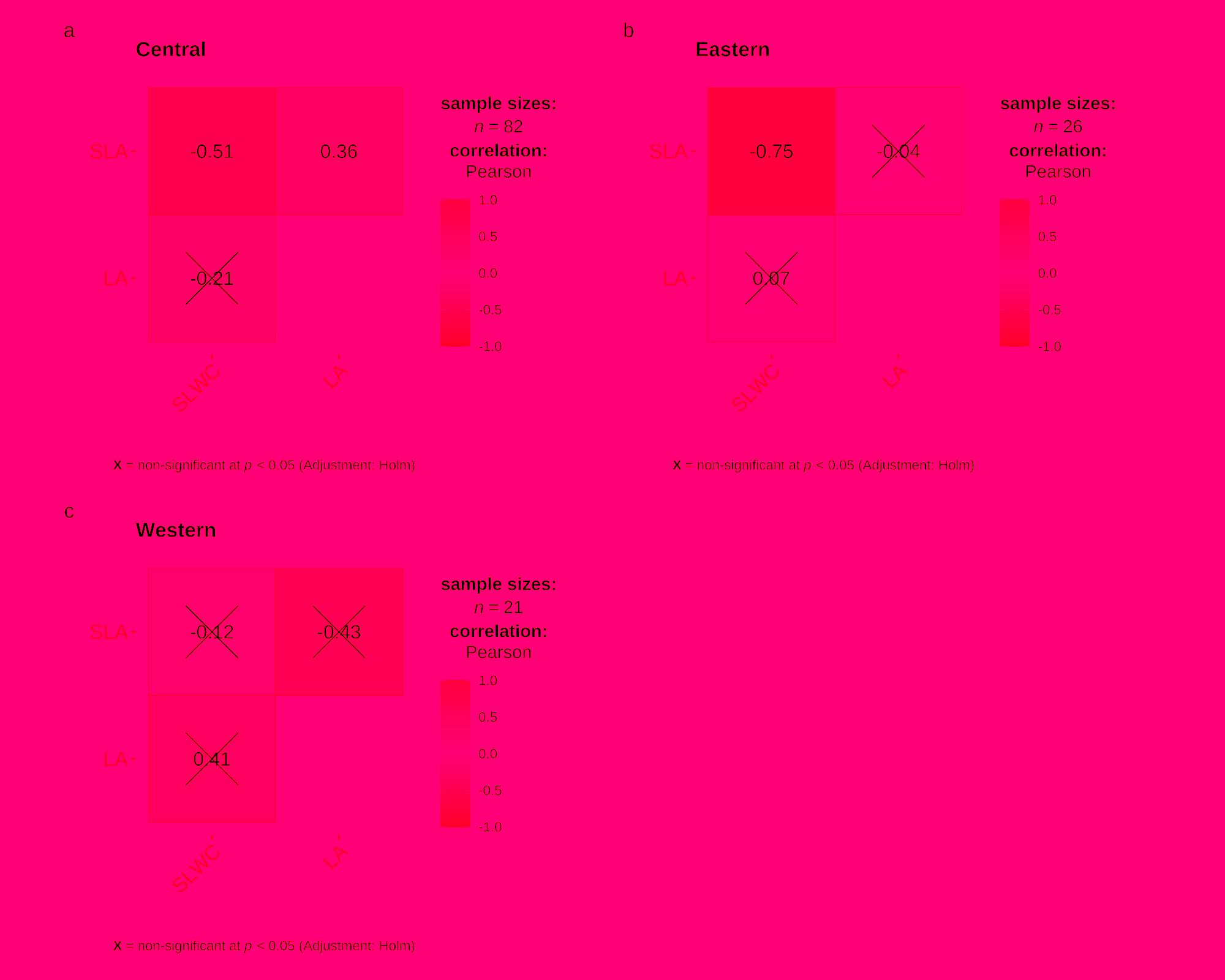

Supplement: Supplementary file 1 [file plants-15-00471-s001.zip › Figure S4.jpg]

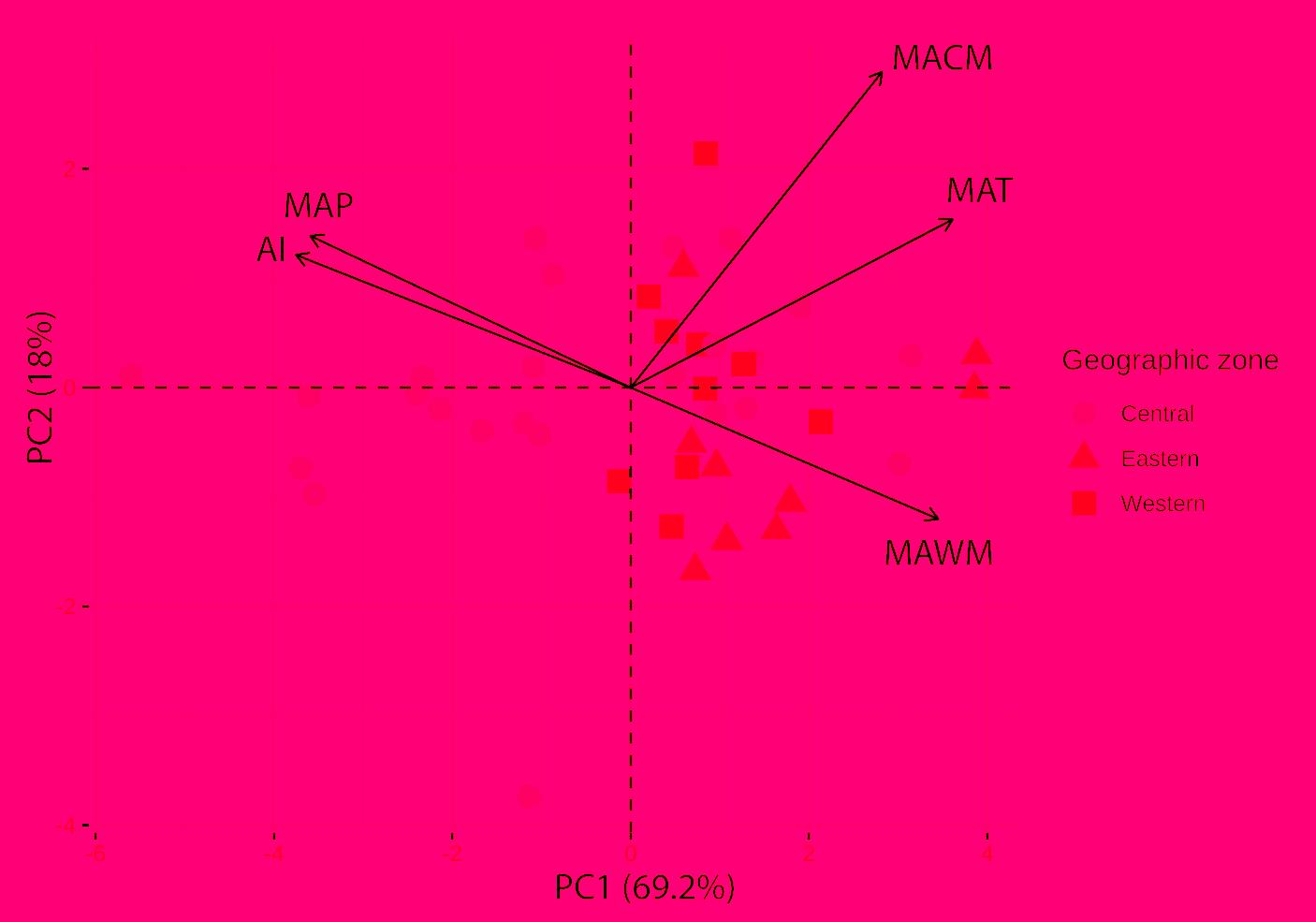

Supplement: Supplementary file 1 [file plants-15-00471-s001.zip › Figure S5.jpg]
